# SIVΔnef vaccination mobilizes systemic and mucosal natural killer cells in Mamu A*01+ macaques

**DOI:** 10.1186/1742-4690-9-S2-O13

**Published:** 2012-09-13

**Authors:** R Reeves, T Evans, J Gillis, M Connole, F Wong, Y Yu, R Johnson

**Affiliations:** 1 NEPRC, Harvard Medical School, Southborough, MA, USA

## Background

Although vaccination with live attenuated SIV is the most effective means of inducing protection against lentiviruses, the immunologic mechanisms responsible remain unclear. Previous studies have yielded conflicting data regarding the role of adaptive immune responses in mediating protection, suggesting that innate immune responses, including natural killer (NK) cells, may play a role.

## Methods

Rhesus macaques were vaccinated with SIVΔnef and challenged with SIVmac239. Cellular dynamics were measured by polychromatic flow cytometry and absolute counts of lymphocytes in blood and rectal biopsies were determined by a bead-based, flow cytometry assay.

## Results

Following SIVΔnef vaccination, circulating NK cells increased 8-fold, peaking at 2 weeks post-vaccination and preceding SIV-specific T cell responses. Furthermore, the gut-homing marker α4β7 was upregulated on circulating NK cells, coinciding with a 2.5-fold increase in NK cells in colorectal tissue. Ki67 expression was upregulated 2- to 5-fold in circulating, lymphoid, and mucosal NK cells. NK cell expansion was also stratified by MHC genotype — Mamu A*01+ macaques showed significant and sustained expansion of NK cells, but not Mamu A*02+ or Mamu B*17+ macaques. SIVΔnef also induced a significant expansion of KIR+ and cytotoxic NK cells in the colorectal mucosa. Interestingly, in vaccinated macaques challenged with wild-type SIV, α4β7 and Ki67 were both upregulated in circulating and mucosal NK cells, even in protected animals and in the absence of an obvious anamnestic response.

## Conclusion

Although current lentiviral vaccines stress the importance of induction of SIV-specific T and B cell responses, NK cells could contribute to protection by inhibiting initial rounds of wild-type virus replication in the mucosa. Our data indicate SIVΔnef induces a robust and sustained expansion of NK cells that traffic to the gut mucosa, and that the protective SIVΔnef NK responses may be modulated, in part, by interaction of Mamu A*01 with an as of yet unidentified KIR.

